# Impact of Metformin on Cancer Biomarkers in Non-Diabetic Cancer Patients: A Systematic Review and Meta-Analysis of Clinical Trials

**DOI:** 10.3390/curroncol28020134

**Published:** 2021-04-06

**Authors:** Tahereh Farkhondeh, Alireza Amirabadizadeh, Hamed Aramjoo, Silvia Llorens, Babak Roshanravan, Farhad Saeedi, Marjan Talebi, Mehdi Shakibaei, Saeed Samarghandian

**Affiliations:** 1 Cardiovascular Diseases Research Center, Birjand University of Medical Sciences, Birjand 9717853577, Iran; farkhondeh2324@gmail.com (T.F.); farhadsaeedi1997@bums.ac.ir (F.S.); 2 Endocrine Research Center, Research Institute for Endocrine Sciences, Shahid Beheshti University of Medical Sciences, Tehran 9717113163, Iran; amirabadiza921@gmail.com; 3 Medical Toxicology and Drug Abuse Research Center (MTDRC), Birjand University of Medical Sciences, Birjand 9717853577, Iran; Hamed.Aramjoo@bums.ac.ir (H.A.); babak.roshanravan@bums.ac.ir (B.R.); 4 Department of Medical Sciences, Faculty of Medicine of Albacete, Centro Regional de Investigaciones Biomédicas (CRIB), University of Castilla-La Mancha, 02008 Albacete, Spain; silvia.llorens@uclm.es; 5 Department of Pharmacognosy, School of Pharmacy, Shahid Beheshti University of Medical Sciences, Tehran 1996835113, Iran; talebi.m@sbmu.ac.ir; 6 Musculoskeletal Research Group and Tumour Biology, Institute of Anatomy, Faculty of Medicine, Ludwig-Maximilians-University Munich, D-80336 Munich, Germany; 7 Noncommunicable Diseases Research Center, Neyshabur University of Medical Sciences, Neyshabur 9318614139, Iran

**Keywords:** metformin, Ki-67, cancer biomarkers, breast cancer, endometrial cancer, non-diabetic

## Abstract

Introduction: Our aim was to investigate and evaluate the influence of metformin on cancer-related biomarkers in clinical trials. Methods: This systematic study was conducted according to Preferred Reporting Items for Systematic Reviews and Meta-Analysis (PRISMA) guidelines. Major databases, including Scopus, Web of Sciences, PubMed, Ovid-Medline, and Cochrane, were systematically reviewed by February 2020. Clinical trials investigating metformin effects on the evaluation of homeostatic models of insulin resistance (HOMA-IR), Ki-67, body mass index (BMI), fasting blood sugar (FBS), and insulin were selected for further analysis. Quality assessment was performed with version 2 of the Cochrane tool for determining the bias risk for randomized trials (RoB 2). Heterogeneity among the included studies was assessed using the Chi-square test. After quality assessment, a random effects model was performed to summarize the data related to insulin, HOMA-IR, Ki-67, and a fixed-effect model for FBS and BMI in a meta-analysis. Results: Nine clinical trials with 716 patients with operable breast and endometrial cancer and 331 with primary breast cancer were involved in the current systematic and meta-analysis study. Systematic findings on the nine publications indicated metformin decreased insulin levels in four studies, FBS in one, BMI in two, Ki-67 in three studies, and HOMA-IR in two study. The pooled analysis indicated that metformin had no significant effect on the following values: insulin (standardized mean differences (SMD) = −0.87, 95% confidence intervals (CI) (−1.93, 0.19), *p* = 0.11), FBS (SMD = −0.18, 95% CI (−0.30, −0.05), *p* = 0.004), HOMA-IR (SMD = −0.17, 95% CI (−0.52, 0.19), *p* = 0.36), and BMI (SMD = −0.13, 95% CI (−0.28, 0.02), *p* = 0.09). Metformin could decrease Ki-67 in patients with operable endometrial cancer versus healthy subjects (SMD = 0.47, 95% CI (−1.82, 2.75), *p* = 30.1). According to Egger’s test, no publication bias was observed for insulin, FBS, BMI, HOMA-IR, and Ki-67. Conclusions: Patients with operable breast and endometrial cancer under metformin therapy showed no significant changes in the investigated metabolic biomarkers in the most of included study. It was also found that metformin could decrease Ki-67 in patients with operable endometrial cancer. In comparison to the results obtained of our meta-analysis, due to the high heterogeneity and bias of the included clinical trials, the present findings could not confirm or reject the efficacy of metformin for patients with breast cancer and endometrial cancer.

## 1. Introduction

The rising incidence of cancer is considered a major threat to population health globally; it is estimated that by 2020, about 16 million cancer patients will be diagnosed as new cases each year [1]. Advances in knowledge for appropriate early detection and treatment with follow-up care, and also the identification of specific cancer biomarkers, may be an effective approach to reducing the burden of cancer. Recently, a number of measurable biomolecules have been defined as biomarkers in cancer, including cyclin D-1, Ki-67 antigen, breast cancer 1 or 2 (BRCA1 or BRCA2), alpha-fetoprotein (AFP), human epidermal growth factor receptor-type2-new (HER2-new) protooncogene, p53 gene, human epididymis protein 4 (HE_4_), and mucin 1 (MUC1) to detect cancer [2]. The focus on combining therapeutics with cancer diagnostics and related biomarkers may play an effective role in the development of cancer medicine.

Metformin, a biguanide anti-diabetic, is usually considered the drug of choice for diabetics. Over the last decade, the extensive use of metformin in human studies has indicated that metformin is useful in reducing mortality and morbidity in diabetic patients caused by cancers such as breast, endometrial, ovarian, prostate, liver, pancreatic, lung, medullary thyroid, gastric, and colon cancer [3,4,5,6]. The metformin impact on cancer cell proliferation has been related to adenosine mono-phosphate-activated protein kinase (AMPK) activation, decreased mTOR signaling, and protein synthesis. Metformin could potentiate the antitumor effect of several MEK inhibitors in lung cancer cells [5,6].

Research (in-vitro) has indicated that metformin inhibits the growth of various human cancer cell lines by controlling the PI3K-AKT-mTOR signaling pathway as well as glucose metabolism [7,8]. Metformin can induce activation of adenosine mono-phosphate-activated protein kinase (AMPK), which leads to suppression of the mTOR pathway [9]. However, due to the use of high doses of metformin in humans, the results of in-vitro studies cannot be attributable to humans and therefore these data do not confirm the beneficial impact of metformin on all types of cancer cells for clinical purposes. Several human studies pointed to the effects of metformin on tumor growth and showed a decrease in the expression of Ki-67 (a biomarker of proliferation) in patients with breast [10,11], prostate [12], and endometrial cancer [13,14,15]. Additionally, metformin regulated some indicators of cancer with poor prognoses such as blood glucose and insulin levels [16,17,18]. There are several debates about the findings of human research on the impact of metformin in cancer patients, as most of them did not compare the metformin group with the placebo or non-drug group.

The aim of the current review was to present the results of clinical trials on the influence of metformin on circulating cancer-related biomarkers and metabolic factors including insulin, fasting blood sugar (FBS), homeostatic model assessment for insulin resistance (HOMA-IR), body mass index (BMI), and Ki-67.

## 2. Methodology

This systematic study was conducted in accordance with the Preferred Reporting Items for Systematic Reviews and Meta-Analysis (PRISMA) recommendations.

### 2.1. Search Strategy

A systematic search of the literature was conducted until February 2020 in the following databases: Ovid-Medline, Scopus, PubMed, Web of Science, and Cochrane. Two groups of search terms were used, which were boolean (AND or OR) without language restrictions. The keywords were as follows:

1. Metformin (metformin, dimethylbiguanidine, dimethylguanylguanidine, glucophage, metformin hydrochloride, and metformin HCl);
2. Cancer and metabolic biomarkers.

The population, intervention, comparison, and outcome (PICO) framework in the present study includes the following information:

P (population): non-diabetic cancer patients;

I (intervention): metformin;

C (comparison): placebo or no drugs;

O (outcome): cancer and metabolic biomarkers.

### 2.2. Inclusion Criteria and Selection Study

Two collaborators (T.F. and H.A.) reviewed the abstract to determine the quality of the studies for the selection of all papers. The abstracts and titles collected from the databases were saved in Endnote, Reference Manager X9, and eliminating duplicates. Two authors then removed unrelated studies by title, abstract, and full text, respectively. Any inconsistencies in the selected studies were discussed and evaluated with third co-workers.

The studies were selected according to the criteria listed below:

(1). The research was based on clinical trial;
(2). Non-diabetic patients with breast or endometrial cancers were selected as participants for the intervention and control groups;
(3). Metformin was administrated for the intervention group;
(4). The endpoint of each study was cancer biomarkers;
(5). The language of articles had to be English.

### 2.3. Exclusion Criteria

(1). Studies with unclear methodology and unclear results were not considered;
(2). Duplicate studies were excluded;
(3). Animals, cohort, case-report, case-control, letters, erratum, and conference papers were excluded.

### 2.4. Data Extraction

Four authors (T.F., H.A., B.R., and F.S.) extracted following data after reading the full text of the selected articles: first author, year of publication, country where RCT was conducted, participants (cancer type, sample size, age, and gender), metformin dose, placebo dose, duration of study, and mean ± standard deviation (SD) or mean ± standard error of mean (SEM) of biomarkers at baseline and after intervention for each study. Discrepancies with the selected studies were clarified through discussion with a third-fourth author.

### 2.5. Quality Assessment

The quality of the studies was independently assessed by three authors (T.F., B.R., and H.A.) using the “Version 2 of the Cochrane risk-of-bias tool for randomized trials (RoB 2)”. Six risks of bias items were individually assessed in the RCTs by answering each question that determines the high (H), unknown (U) or low risk (L) of bias. Performance bias was determined by two domains including random housing and blinding. Detection bias were done according to the random outcome assessment and blinding outcome assessment. Attrition bias was revealed according to the incomplete outcome data. Reporting bias considered how selective outcome reporting was and what was found. Other sources of bias in result and method sections consisted of any important concerns about bias not covered by other domains in the tool. High risk of bias indicates low quality of articles. Low risk of bias indicates high quality of articles. Unclear risk of bias indicates that the limited information restricted correct judgment to identify low or high quality of the article.

### 2.6. Statistical Analysis

The heterogeneity of the selected articles was evaluated using the “Chi-square test”. The I^2^ statistic was used to examine the total variation percentage between articles. To estimate the pooled effect, a model with random-effects was used when I^2^ was higher than 50% and a model with fixed-effects model if I^2^ was lower than 50%. The pooled effect of metformin on cancer biomarkers was indicated as effect size, odds ratio (OR), and standardized mean differences (SMD) with 95% confidence intervals (CI). Overall results showed a significant difference between the two groups when the level of *p*-value was less than 0.05 (*p* < 0.05). The Egger regression test was used to determine the publication bias. A sensitivity analysis was performed to determine the effect of each included study on the size of the pooled effect. The meta-analysis in this study was performed with “STATA 13 software” (R3.3.1, University of Auckland, Auckland, New Zealand).

## 3. Results

### 3.1. Study Selection

**Figure 1:**
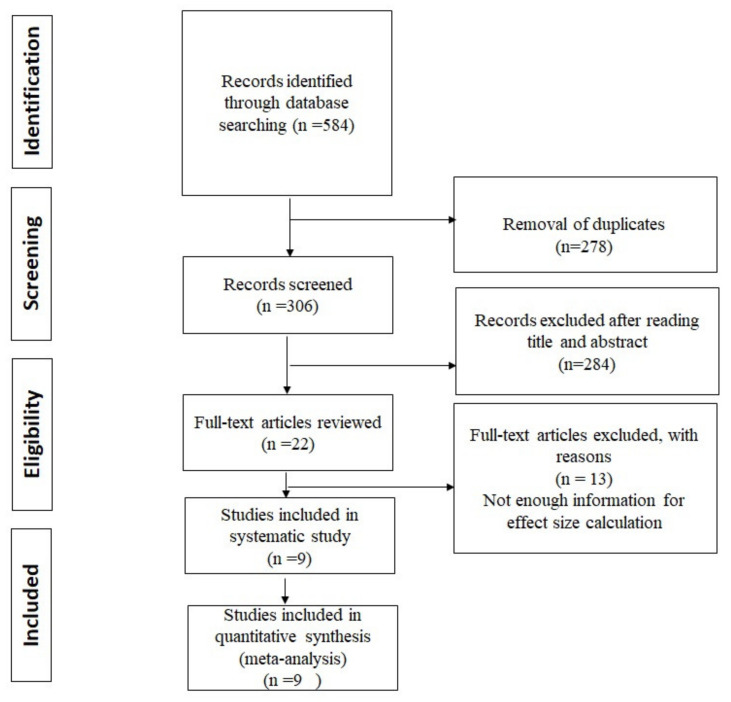
Preferred Reporting Items for Systematic Reviews and Meta-Analysis (PRISMA) Flowchart of the literature search of the included studies.

After an initial search with selected keywords for this study, we received 306 articles. Following the eliminating duplicates, 194 studies were evaluated according to the title and abstract and 22 of these were selected for a complete evaluation of eligibility for funding. Nine RCTs matched our inclusion criteria and were selected in the present systematic and high-quality study (Figure 1).

### 3.2. Features of the Study

Table 1 indicates the characteristics of the included studies. The selected studies were performed in Italy [10,19], USA [20,21], Great Britain [22,23], Canada [13,24], and Spain [25]. The sample size was 1047 participants in metformin treatment and control groups, and their age ranged from 42 to 77 years. The studies were performed between 2012 and 2019. The sample size was ranged from 21 to 507 for groups of included studies.

The metformin dose ranged from 500 to 2000 mg/day. The duration of studies varied between 2 weeks and 42 weeks. Seven studies assessed the impact of metformin compared to placebo prescribed to breast cancer, and two studies evaluated the impact of metformin in a patient with endometrial cancer. Eight studies indicated the effect of metformin on the insulin levels [10,13,20,21,22,23,24,25], six studies on FBS [10,20,21,23,24,25], five on HOMA-IR [19,20,23,24,25], eight on BMI [10,13,19,20,21,22,23,24], and seven on Ki-67 [10,13,19,20,22,23,24].

### 3.3. Systematic Findings

Our systematic findings indicated that metformin could decrease insulin levels in 3 studies [13,21,24] and had no effect on other four studies. FBS was decreased in patients under metformin therapy in one study [24] and did not change in other study. Three studies indicated that metformin reduced BMI in patients [10,20,24] and could not change BMI in other four studies. Ki-67 was decreased in participants under metformin therapy in 4 studies [13,22,23,24] and did not change in the other three studies [10,19,20]. Metformin decreased the HOMA-IR in two studies [20,24] and had not effect in other studies.

### 3.4. Risk of Bias Assessment

The quality of the evaluation of the articles has been shown in Table 2. Five RCTs reported information on the generation of random sequence with low bias [10,13,19,20,22,24,25]. Four studies did not show any concealment of the assignment [21,22,23,24]. Six studies focused on the type of blinding [10,19,20,22,24,25]. Four studies indicated their findings with a low risk of bias of the incomplete outcome [10,13,21,23]. Four studies also showed a low risk of bias in selective reporting [20,21,24,25]. In general, the studies were of different quality in their structure, and the detailed examination did not show high risk of bias.

### 3.5. Results of the Meta-Analysis

#### 3.5.1. Impact of Metformin on Insulin Levels

**Figure 2:**
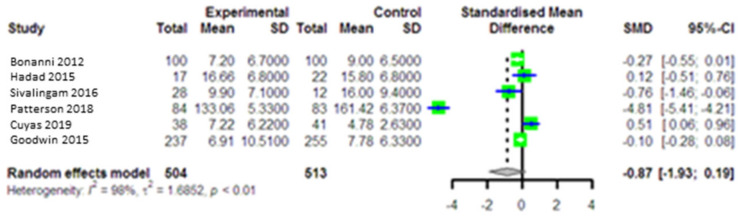
Forest plot of the meta-analysis comparing plasma insulin levels between intervention and control groups. SMD: standardized mean difference, SD: standard deviation, CI: confidence intervals.

A pooled analysis of four studies involving 1017 participants (intervention group = 6 and comparison group = 6) indicated that metformin was unable to significantly lower insulin levels in the intervention group compared to the comparison group (SMD = −0.87, 95% CI (−1.93, 0.19), *p* = 0.11) (Figure 2). For insulin, a high heterogeneity (I^2^ = 98.0, *p* = 0.14), a very low effect of individual studies (SMD: −2.12, 0.25), and no publication bias (*p* = 0.72) was found.

#### 3.5.2. Impact of Metformin on Fasting Blood Glucose (FBS)

**Figure 3:**
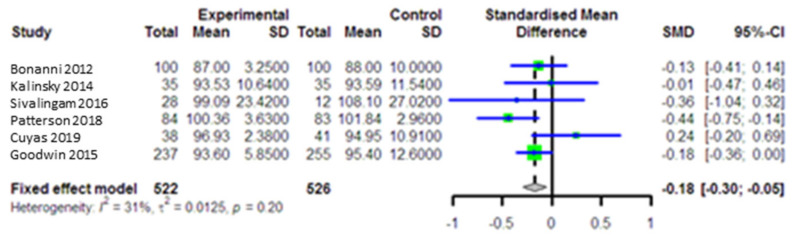
Forest plot of the meta-analysis to compare plasma fasting blood sugar (FBS) levels between intervention and control groups.

Five studies on 1048 participants (intervention group = 522 and comparison group = 526) evaluated the impact of metformin on FBS as an endpoint. Pooled data using a randomized effect model indicated that metformin could significantly reduce FBS levels (SMD = −0.18, 95% CI (−0.30, −0.05), *p* = 0.004) in intervention group compared to the control group (Figure 3), with pronounced heterogeneity between studies (I^2^ = 31.0, *p* = 0.20) and without publication bias (*p* = 0.76). The sensitivity assessment indicated that the effect of the individual studies was very small (SMD: −0.17–−0.01).

#### 3.5.3. Impact of Metformin on HOMA-IR

**Figure 4:**
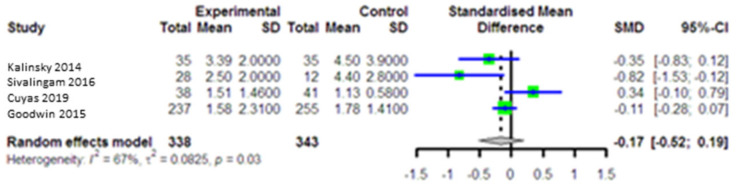
Forest plot of the meta-analysis to compare homeostatic models of insulin resistance (HOMA-IR) between intervention and control groups.

In four studies with 681 patients (intervention group = 338 and comparison group = 343), the HOMA-IR modification in the intervention group was compared with the comparison group. The pooled analysis indicated that the combined effect size (SMD = −0.17, 95% CI (−0.52, 0.19), *p* = 0.36) (Figure 4) with high heterogeneity across the studies (I^2^ = 67.0, *p* = 0.03) was very small, the effect of individual SMD: −0.21, 0.09. According to Egger’s test, no publication bias was observed for HOMA-IR (*p* = 0.56).

#### 3.5.4. Impact of Metformin on Body Mass Index (BMI)

**Figure 5:**
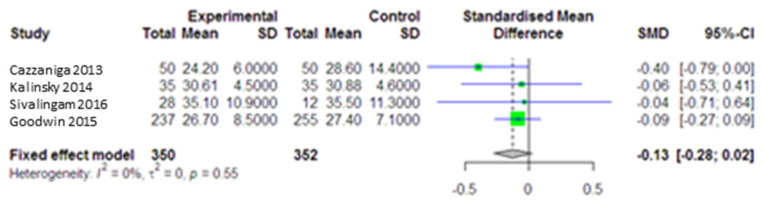
Forest plot of the meta-analysis to compare BMI between intervention and control groups.

The pooled data from three studies with 702 cancer patients (intervention group = 4 and comparison group = 4) showed that metformin could not significantly reduce BMI (SMD = −0.13, 95% CI (−0.28, 0.02), *p* = 0.09) in the intervention group compared the control group (Figure 5). No heterogeneity was observed in the studies assessing the effect of metformin on BMI (I^2^ = 0.0, *p* = 0.55). The ssensitivity analysis indicated that the effect of the individual studies was very small (SMD: −0.06, 0.27). A publication bias was not reported.

#### 3.5.5. Impact of Metformin on the Ki-67 Level in Plasma

**Figure 6:**
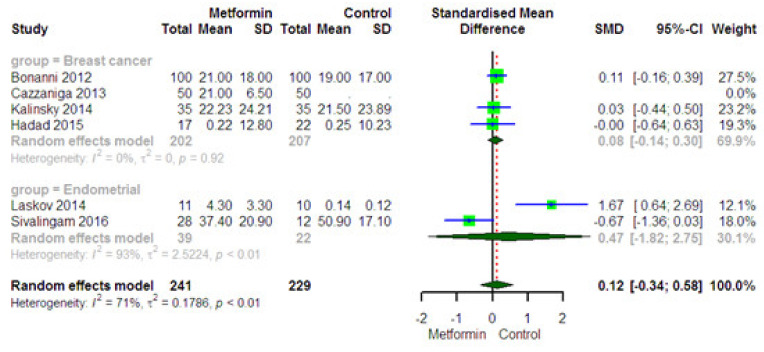
Forest plot of the meta-analysis to compare plasma Ki-67 levels between intervention and control groups.

Four studies provided 202 operable breast cancer patients and 207 healthy people for Ki-67 as a specific endpoint for cancer. The combined result of the randomized outcome models indicated that metformin had no significant effect on Ki-67 concentration (SMD = 0.08, 95% CI (−0.14, 0.30), 69.9%) in the intervention group versus with the control group. There was no heterogeneity between the studies assessing the effect of metformin on Ki-67 (I^2^ = 0%, *p* = 0.92). Two studies provided 39 operable endometrial cancer patients and 22 healthy people for Ki-67 as a specific endpoint for cancer. The combined result of the randomized outcome models indicated that metformin decreased Ki-67 concentration (SMD = 0.47, 95% CI (−1.82, 2.75), 30.1%) in the intervention group versus with the control group. The heterogeneity between the studies assessing the effect of metformin on Ki-67 was high (I^2^ = 93, *p* < 0.01) (Figure 6). The sensitivity analysis indicated that the effect of the individual studies was very small (SMD: 0.07, 0.16). According to Egger’s test, no publication bias was observed for Ki-67 (*p* = 0.17).

## 4. Discussion

The present study aimed to demonstrate the scientific evidence on the impact of metformin on biomarkers that could be linked to the occurrence of cancer. There was evidence from nine RCTs investigating the impact of metformin in patients with breast and endometrial and non-diabetic cancer. Systematic findings on the nine publications indicated metformin decreased insulin levels in three studies, FBS in one, BMI in three, Ki-67 in three, and HOMA-IR in one.

Our pooled analysis findings showed that metformin therapy did not have a significant effect on measured biomarkers such as insulin, glucose, HOMA-IR, or BMI. We also found that metformin could decrease Ki-67 in patients with operable endometrial cancer. However, due to the high heterogeneity of the included studies, the data from the current study should be interpreted with caution.

It is important to consider that the high heterogeneity among studies evaluating the effect of metformin on insulin, glucose, HOMA-IR, BMI, and Ki-67 affected our data. The RCTs included varied in study population characteristics such as overweight/obese patients, not-overweight/obese patients, patients with two cancer, and patients with stage 1–3 cancer, as well as in sample size, duration and dose of metformin treatment, and adjustment for confounders.

Our findings are similar to data from a meta-analysis of observational studies conducted by Tang et al. (2018), which examined the impact of metformin on breast cancer incidence and mortality [26]. They found no association between metformin therapy in diabetic breast cancer patients and interpreted that the bias of observational studies prevented a safe conclusion. They suggested that it was necessary to design RCTs to prove the exact association between metformin and breast cancer.

Three of the RCTs included in the current meta-analysis [10,19,20] did not report a significant reduction effect of metformin on Ki-67 in non-diabetic breast cancer patients compared to their controls. This is in contrast to the results of a meta-analysis conducted by Rahmani et al. (2019) on nine RCTs [27]. They reported that metformin decreased insulin, glucose, leptin, C-reactive protein (CRP), HOMA-IR, and Ki-67 levels, which attenuated the outcome in breast cancer patients. The difference between our findings and the study by Rahmani et al. (2019) is related to another article included in two meta-analyses. In two included articles in the studies of Rahmani et al. (2019), those of Laskov et al. (2014), and Sivalingam et al. (2016)), it was reported that metformin decreased circulating insulin, glucose, and Ki-67 levels in cancer patients who did not meet our inclusion criteria such as English language and RCTs restriction. Due to the limited number of available studies on the effect of metformin on the Ki-67 biomarker in the endometrial cancer patient, no subgroup analysis was performed.

Ki-67 is a deterministic prognostic biomarker for the detection of breast cancer [28,29,30], but there are few documents on its use as a diagnostic biomarker for endometrial cancer. It was found that high-stage cancer had higher levels of Ki-67. Several investigations reported an association between high levels of Ki-67, insulin, and glucose, and a poor prognosis in endometrial cancer [31,32,33,34]. In two RCTs [13,23], metformin was found to significantly decrease the expression of Ki-67 in patients with endometrial cancer. Metformin changed in phosphorylated mTOR proteins and serum markers of insulin resistance. It was suggested that metformin could decrease Ki-67 expression only in patients with insulin resistance.

In cancerous cells, the levels of blood circulating insulin and IGF increases, thereby the growth of these cells is induced. Metformin can decrease hepatic gluconeogenesis and lipogenesis and also elevate fatty acid oxidation, insulin sensitivity, and inhibiting gluconeogenesis. This ameliorates blood glucose control, reducing food consumption and intestinal glucose absorption [28]. However, the mechanisms related to its effect on cancer biomarkers have not fully understood. Metformin could decrease Ki-67 in patients with operable endometrial cancer prior to surgery and increase apoptosis, leading to inhibit cancer progression. For breast cancer patients, it seems that metformin may be effective in early stage. However, several factors such as genetic polymorphisms may cause various response to metformin in cancer patients. It is necessary to find target populations for its use.

Our finding did not confirm or reject the protective effect of metformin in woman with endometrial and breast cancer, as most RCTs in our meta-analysis did not consider insulin resistance as the most important confounding factor.

Our meta-analysis study has one advantage, including the use of RCTs to assess the association between the metformin therapy and biomarkers changes.

However, this study has several limitations. First, high heterogeneity of the articles and the bias of the publications affected our data. Second, all included studies had a small sample size and poor quality. Third, the small number of selected studies limited us to performing the subgroup analysis for duration and dose of intervention of metformin effects on cancer-related biomarkers and also age of subjects. Fourth, we could not find sources of heterogeneity between included publications in this meta-analysis.

## 5. Conclusions

Present systematic findings indicate that metformin could decrease insulin levels, FBS, BMI, Ki-67, and HOMA-IR in non-diabetic patients with endometrial and breast cancer in some studies. The reduction of the biomarkers related to endometrial and breast cancer was observed in our systematic findings when the duration of metformin intervention was between 2 to 24 weeks. Although metformin has been introduced as a new drug for several types of cancer in recent years, our meta-analysis results show that metformin therapy is not able to influence insulin, FBS, HOMA-IR, and BMI levels in endometrial and breast cancer patients. However, we found that metformin could decrease Ki-67 in operable endometrial patients. Altogether, the quality of the scientific documents from this study was negligible and our results should be interpreted with caution. We recommend the design of several large RCTs with appropriate sample sizes to perform a definitive conclusion.

## Figures and Tables

**Table 1:** Characteristics of the studies included in the meta-analysis and systematic review of metformin effects on cancer-related biomarkers.

First Author Year Country	Study Design	Sample Size	Subjects	Age Range Duration	Intervention Dosage	Control Dosage	Result	Reference
Bonanni 2012 Italy	RCT	200	Operable Breast Cancer	42–634 Weeks	1700 mg/day	1700 mg/day	Insulin (↔) FBS (↔) BMI (Reduced) Ki-67 (↔)	[10]
Cazzaniga 2013 Italy	RCT	100	Operable Breast Cancer	45–624 weeks	1700 mg/day	1700 mg/day	HOMA-IR (↔) BMI (↔) Ki-67 (↔)	[19]
Laskov 2014 Canada	RCT	21	Operable Endometrial Cancer	49–765 Weeks	1500 mg/day	No Drug	Insulin (Reduced) BMI (↔) Ki-67 (Reduced)	[13]
Kalinsky 2014 USA	RCT	70	Operable Breast Cancer	45.3–67.32 Weeks	1500 mg/day	No Drug	Insulin (Reduced) FBS (↔) HOMA-IR (Reduced) BMI (Reduced) Ki-67 (↔)	[20]
Hadad 2015 UK	RCT	39	Operable Breast Cancer	- 2 Weeks	500 mg/day, 2000 mg/day	No Drug	Insulin (↔) BMI (↔) Ki-67 (Reduced)	[22]
Goodwin 2015 Canada	RCT	507	Primary Breast Cancer	- 42 weeks	850 mg/dsy	850 mg/day	Insulin (Reduced) FBS (Reduced) HOMA-IR (Reduced) BMI (Reduced) Ki-67 (Reduced)	[24]
Sivalingam 2016 UK	RCT	40	Operable Endometrial Cancer	54.7–774 Weeks	1700 mg/day	No Drug	Insulin (↔) FBS (↔) HOMA-IR (↔) BMI (↔) Ki-67 (Reduced)	[23]
Patterson 2018 USA	RCT	167	Operable Breast Cancer	55.7–70.524 Weeks	500 mg/day, 1500 mg/day, 2000 mg/day	500 mg/day, 2000 mg/day	Insulin (Reduced) FBS (↔) BMI (↔)	[21]
Cuyas 2019 Spain	RCT	79	Operable Breast Cancer	- 24 Weeks	850 mg/day	850 mg/day	Insulin (↔) FBS (↔) HOMA-IR (↔)	[25]

↔: No change.

**Table 2:** Quality of the studies.

Study	Random Sequence	Allocation Concealment	Blinding	Incomplete Outcome Data	Selective Reporting	Other Bias	Reference
Hadad 2015	L	L	L	H	U	L	[22]
Goodwin 2015	L	L	L	U	L	U	[24]
Sivalingam 2016	H	L	U	L	H	U	[23]
Cazzaniga 2013	L	U	L	H	U	L	[19]
Cuyas 2019	U	H	L	U	L	L	[25]
Bonanni 2012	L	U	L	L	U	H	[10]
Laskov 2014	L	H	H	L	U	L	[13]
Kalinsky 2014	H	U	L	U	L	L	[20]
Patterson 2018	U	L	H	L	L	H	[21]

## Data Availability

The data presented in this study are available in the main text.
